# Relationship between biofilm formation and antibiotic resistance of *Klebsiella pneumoniae* and updates on antibiofilm therapeutic strategies

**DOI:** 10.3389/fcimb.2024.1324895

**Published:** 2024-02-23

**Authors:** Lifeng Li, Xueyan Gao, Mingchao Li, Yuchun Liu, Jiayue Ma, Xiaolei Wang, Zhidan Yu, Weyland Cheng, Wancun Zhang, Huiqing Sun, Xiaorui Song, Zhaobao Wang

**Affiliations:** ^1^ Henan International Joint Laboratory of Children’s Infectious Diseases, Department of Neonatology, Children’s Hospital Affiliated to Zhengzhou University, Henan Children’s Hospital, Zhengzhou Children’s Hospital, Zhengzhou, China; ^2^ Department of Epidemiology, College of Public Health, Zhengzhou University, Zhengzhou, China; ^3^ Medical Science and Technology Innovation Center, Shandong First Medical University & Shandong Academy of Medical Sciences, Jinan, China; ^4^ State Key Laboratory of Microbial Technology, Shandong University, Qingdao, China; ^5^ State Key Laboratory Cultivation Base, Shandong Provincial Key Laboratory of Ophthalmology, Eye Institute of Shandong First Medical University, Qingdao, China; ^6^ Energy-rich Compounds Production by Photosynthetic Carbon Fixation Research Center, Shandong Key Lab of Applied Mycology, College of Life Sciences, Qingdao Agricultural University, Qingdao, China

**Keywords:** *Klebsiella pneumoniae*, biofilm formation, antibiotic resistance, therapeutic strategies, regulation mechanisms

## Abstract

*Klebsiella pneumoniae* is a Gram-negative bacterium within the *Enterobacteriaceae* family that can cause multiple systemic infections, such as respiratory, blood, liver abscesses and urinary systems. Antibiotic resistance is a global health threat and *K. pneumoniae* warrants special attention due to its resistance to most modern day antibiotics. Biofilm formation is a critical obstruction that enhances the antibiotic resistance of *K. pneumoniae*. However, knowledge on the molecular mechanisms of biofilm formation and its relation with antibiotic resistance in *K. pneumoniae* is limited. Understanding the molecular mechanisms of biofilm formation and its correlation with antibiotic resistance is crucial for providing insight for the design of new drugs to control and treat biofilm-related infections. In this review, we summarize recent advances in genes contributing to the biofilm formation of *K. pneumoniae*, new progress on the relationship between biofilm formation and antibiotic resistance, and new therapeutic strategies targeting biofilms. Finally, we discuss future research directions that target biofilm formation and antibiotic resistance of this priority pathogen.

## 1 Introduction

Due to the widespread use of antibiotics around the world, there has been an increasing development of bacterial resistance to antibiotics. The rising frequent acquisition of functional genes through mobile components has given rise to increased drug resistance and virulence of *Klebsiella pneumoniae* (*K. pneumoniae*) (Cai et al., 2022). *K. pneumoniae* is a Gram-negative bacterium of the *Enterobacteriaceae* family that is widely distributed in nature, and can colonize the intestinal mucosa, skin and nasopharynx of the host as a symbiotic bacteria (Podschun and Ullmann, 1998). It can cause a wide range of infections, such as respiratory system infection, bloodstream infection, liver abscess and urinary tract infection (Li et al., 2023).

Antimicrobial-resistant ESKAPE (*Enterococcus faecium*, *Staphylococcus aureus*, *Klebsiella pneumoniae*, *Acinetobacter baumannii*, *Pseudomonas aeruginosa* and *Enterobacter species*) pathogens are serious health threat, which necessitates specific attention towards the development of novel therapeutics. *K. pneumoniae* is a member of the ESKAPE pathogen group due to its ability to escape the inhibitory effects of antibiotics (Saha et al., 2023). Antibiotic resistance of this bacterium is further enhanced by biofilm formation.

Biofilm refers to a structured community of microorganisms wrapped in extracellular polymeric substance (EPS) (Cruz et al., 2021). EPS accounts for 90% of the biofilm and is mainly composed of polysaccharides, proteins and DNA (Cruz et al., 2021). Compared to planktonic cells, bacteria within the biofilms are 1000 times more resistant to antibiotics due to the presence of thick EPS layers, enhanced expression of efflux pumps, and the presence of persistent cells (Singh et al., 2022). Currently, 60-80% bacterial infections are associated with biofilm formation (Ribeiro et al., 2016). Biofilms can protect the pathogen from host immune responses and the antipathogenic effects of antibiotics, thereby improving antibiotic resistance and survivability of the bacteria, in addition to increasing the difficulty of treating the ensuing disease. Nunez et al. found that biofilm formation contributed to the survival of bacteria on hospital surfaces, which are highly resistant to desiccation, benzalkonium chloride disinfection and UV radiation, making the treatment of infected biomaterials and tissue surfaces difficult for clinicians (Nunez et al., 2023). Therefore, analyzing critical genes and regulation mechanisms of biofilm formation is of great significance for exploring new targets for the prevention and control of biofilm-related infections.

In the present review, we analyzed research progress on the formation of biofilms by *K. pneumoniae* to provide insight for the control of biofilm-related infections. Specifically, we summarized *K. pneumoniae* biofilm formation and the genes involved, its association with antibiotic resistance, as well as novel approaches for the treatment of biofilm-related infections.

## 2 Stages of biofilm formation

**Figure 1:**
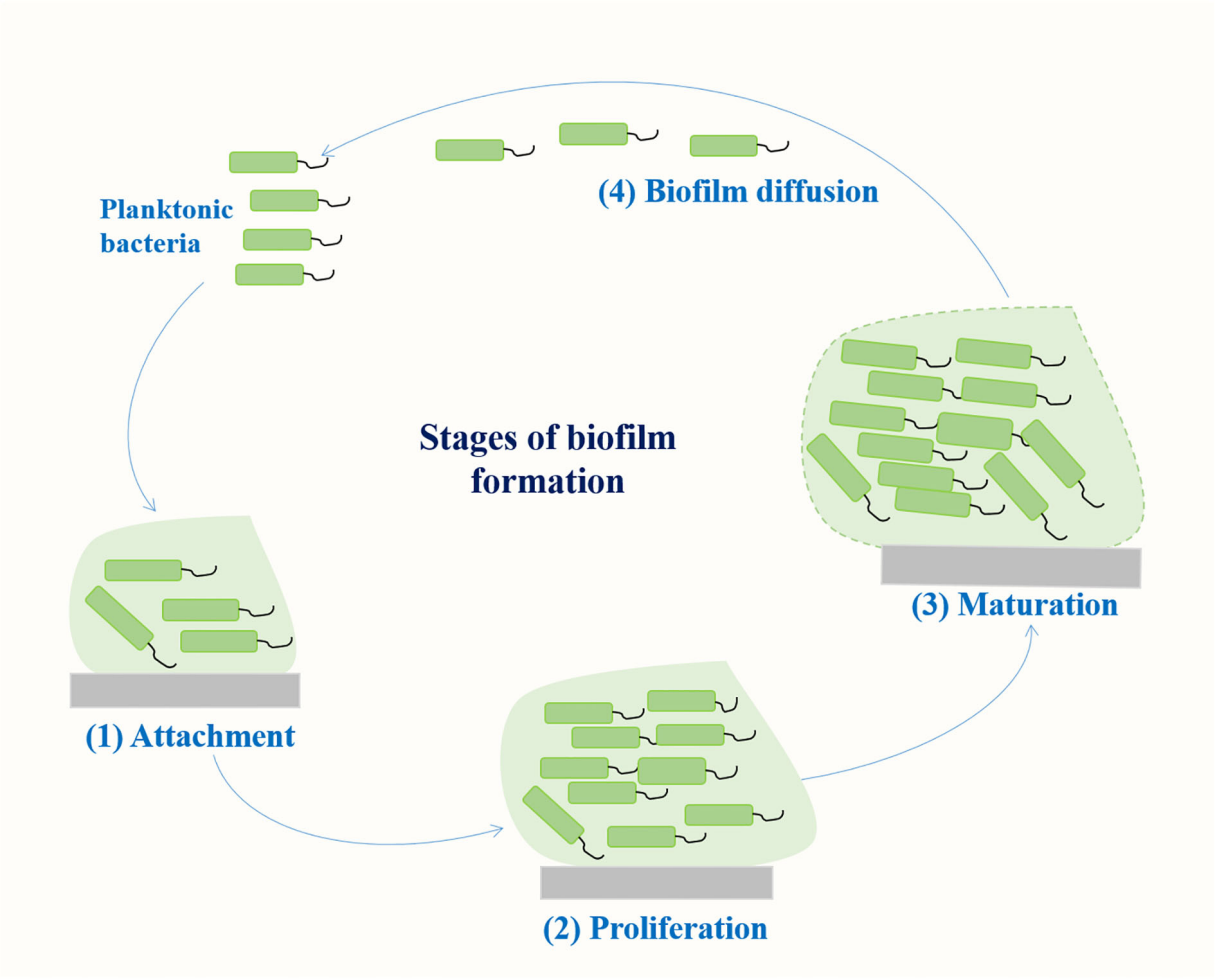
Stages of biofilm formation.

**Figure 2:**
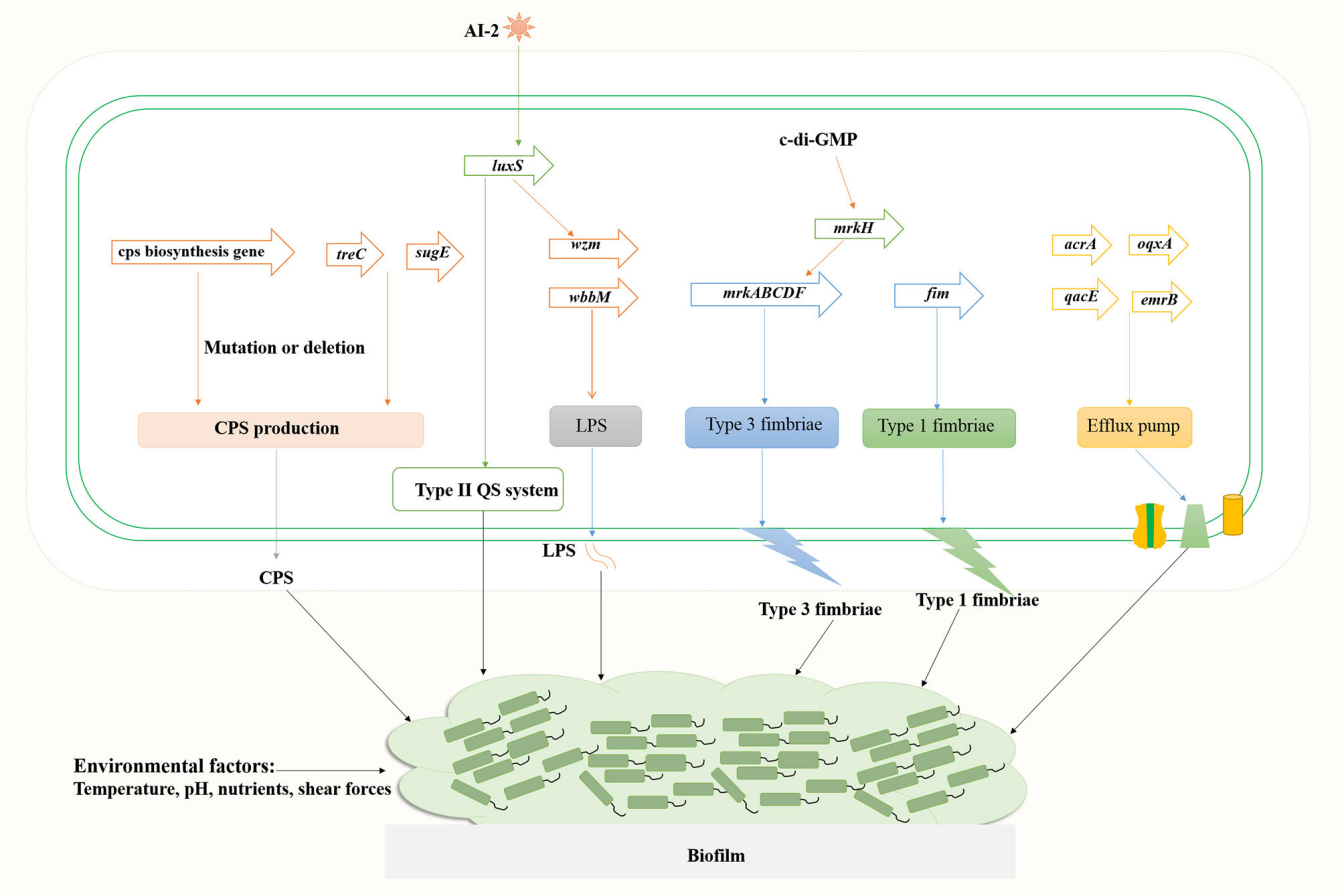
Genes involved in the regulation of *K. pneumoniae* biofilm formation.

Biofilm is a community of microorganisms attached to a living or non-living surface, which can be found on the skin, mucosa, and teeth of human body, and are commonly found in implantable medical devices, such as central venous catheters or artificial hip or knee joints (Varma et al., 2023). A biofilm results from a series of events that commence with the contact of planktonic bacteria with a surface. The transformation of bacteria from plankton state to biofilm state is a complex process regulated by both genetic and environmental factors. Genes involved in *K. pneumoniae* biofilm formation mainly include fimbriae, polysaccharides, quorum sensing (QS) system, efflux pump, etc (Schroll et al., 2010; Chen et al., 2020; Guerra et al., 2022). The formation of biofilm mainly includes four stages (**Figure 1**): (1) reversible bacterial attachment; (2) adhesion and proliferation; (3) biofilm maturation; and (4) biofilm diffusion (Alav et al., 2018; Wang et al., 2020). Planktonic bacteria respond to various environmental signals and attach to surfaces to form colonies. After the formation of colonies, the bacteria produces a matrix composed of polysaccharides, proteins and lipids, and the colony matures into larger colonies. At this stage, the bacteria can either separate from the biofilm and return to a floating state or form a biofilm on the surface. The percentage of protein, sugar and eDNA in biofilm matrices of *K. pneumoniae* isolates varies among isolates from different infection sites. Higher amounts of protein were detected in the biofilms of blood and pus isolates, whereas for the biofilms of urine isolates, a higher amount of sugar was detected (Singh et al., 2019). Several important virulence factors such as capsular polysaccharides and fimbriae also contribute to biofilm formation in *K. pneumoniae* (Balestrino et al., 2008; Guerra et al., 2022) (**Figure 2**).

*K. pneumoniae* strains produce different types of exopolysaccharides, which corresponds to different capsular antigenic serotypes. Transposon insertion within ORF12 of the K2 gene cluster can lead to the reduction of capsule production and a significant defect in biofilm formation on a surface coated with human extracellular matrix material (Boddicker et al., 2006). Gene disruption and microscopic analyses indicated that LPS is involved in initial adhesion on both glass and polyvinyl-chloride (PVC), whereas the capsule is required for the appropriate initial attachment and biofilm maturation (Balestrino et al., 2008). Genes associated with biofilm formation include ORF4 (*wza* homologous, transport of capsular polysaccharides), ORF14 (glycosyl transferase, capsule biosynthesis), *wzm* (LPS transport pathway) and *wbbM* (LPS biosynthesis), etc. Mutations in *wza* and ORF14 resulted in decreased adherence ability, and transposon insertions within the CPS loci *wza* and *wzc* resulted in a deficiency in biofilm formation. Wu et al. found that the polysaccharide production related genes *treC* (an enzyme that splits trehalose-6-phosphate into glucose and glucose-6-phosphate) and *sugE* (encode an inner-membrane protein with a very short tail facing the cytoplasm) affect biofilm formation by modulating capsular polysaccharide production in *K. pneumoniae*, causing pyogenic liver abscess (Wu et al., 2011). Results of *in vivo* competition analysis showed that the *treC* mutant had weakened intestinal colonization ability.

The function of polysaccharides, such as CPS and LPS, in the composition of biofilm matrices includes, but is not limited to, being a physical component that envelops bacterial cells and allows the penetration of active small molecules (Bellich et al., 2018). The chemical structure of biofilm polysaccharides has high variability and a single bacterial species can produce many different polysaccharides, indicating that polysaccharides have specific biological roles in bacterial biofilms. The polysaccharide capsule can influence initial surface adhesion and biofilm maturation of *K. pneumoniae*. Expression of the capsule biosynthesis gene *wcaG* facilitated biofilm formation in *K. pneumoniae* and *wcaG* silencing led to reduced biofilm formation (Zheng et al., 2018).

Most *K. pneumoniae* isolates express two types of fimbrial adhesins, type 1 and type 3 fimbriae. Analysis of type 1 fimbriae mutant (C3091Δ*fim*), type 3 fimbriae mutant (C3091Δ*mrk*), and double mutant (C3091Δ*fim*Δ*mrk*) using a biofilm hydrodynamic flow chamber experiment identified that type 3 fimbriae, but not type 1 fimbriae, are important for the bacterial attachment of biofilm formation in *K. pneumoniae* (Schroll et al., 2010). However, significant attenuation in catheter biofilm formation in the absence of either type 1 fimbriae or type 3 fimbriae indicated that both fimbrial types play a role in bacterial colonization on catheter surfaces or in biofilm formation (Stahlhut et al., 2012). Type 3 fimbriae formed by the mannose-resistant *Klebsiella*-like (MR/K) hemagglutinins (Mrk proteins) are encoded by *mrkABCDF* operon (Wilksch et al., 2011). Mutants of the *mrkABCDF* genes were severely defective in biofilm formation (Wilksch et al., 2011). The intracellular cyclic diguanylate (c-di-GMP) is a second signal messenger in bacteria, which is involved in bacterial biofilm formation. The c-di-GMP-dependent transcriptional activator MrkH binds to the MrkH box (TATCAA) upstream of the *mrkABCDF* operon and activates the expression type 3 fimbriae (Tan et al., 2015).

The quorum sensing system promotes biofilm maturation through sensing bacterial signal molecules and coordinating bacterial population density (**Figure 2**). Type II QS is a *luxS-*dependent bacterial communication system and has a sense autoinducer-2 (AI-2) signal in *K. pneumoniae* (Balestrino et al., 2005; Chen et al., 2020). Increased expression of AI-2 was detected in the early stationary phase when the media was supplemented with different carbohydrates (glucose, sucrose or glycerol) (Chen et al., 2020). Deletion of *luxS* gene led to a changed biofilm architecture with less surface coverage and reduced macrocolony formation. Decreased expression of lipopolysaccharide biosynthesis gene *wzm* (2.7-fold) and upregulated expression of *pgaA* encoding a porin for poly-β−1,6-N-acetyl-d-glucosamine (PNAG) were detected, whereas the expression of type 3 fimbriae biosynthesis *mrkA* gene was unaffected (Balestrino et al., 2005; Chen et al., 2020).

Efflux pump is also a causing agent of biofilm formation in *K. pneumoniae* (**Figure 2**). Five main families of bacterial efflux pumps have been reported including Resistance-nodulation-division (RND), ATP-binding cassette (ABC), Major facilitator superfamily (MFS), Small multi-antibiotic resistance (SMR) and Multidrug and toxic compound extrusion (MATE) family/superfamily (Li et al., 2015). Efflux pumps may play multiple roles in biofilm formation: (i) Efflux EPS and/or QS molecules to promote biofilm matrix formation; (ii) Regulate genes involved in biofilm formation; (iii) Efflux harmful molecules, such as antibiotics and metabolic intermediates; (iv) Affect aggregation by promoting or preventing adhesion to surfaces and other cells (Alav et al., 2018). Efflux pumps AcrA and OqxA belong to the RND family, whereas QacEΔ1 (a deletion form of QacE) and EmrB belong to the SMR and MFS family, respectively. Tang et al. analyzed the distribution of efflux pump genes in multidrug resistance (MDR) *K. pneumoniae* strains, and the positive rate of *emrB* was 89.29%, followed by 78.57% for *oqxA*, 39.29% for *qacEΔ1* and 35.71% for *acrA* (Tang et al., 2020). Relative transcription levels of *acrA*, *emrB*, *oqxA*, and *qacEΔ1* in *K. pneumoniae* biofilms were significantly upregulated compared to the planktonic cells. Efflux pump inhibitor carbonyl cyanide m-chlorophenyl hydrazine (CCCP) inhibited biofilms in a dose-dependent manner. Kvist et al. reported that efflux pump inhibitors (EPIs) significantly affected the biofilm formation of uropathogenic *Klebsiella* sp., and combinations of different types of EPIs resulted in close to 100% inhibition of the bacterial biofilm formation, whereas the addition of EPI to biofilms of *K. pneumoniae* significantly increased the sensitivity of the strain to tetracycline (Kvist et al., 2008).

Environmental factors affect biofilm formation as well (**Figure 2**). Shear forces can influence biofilm formation as bacteria has been shown to form relatively flat biofilms under higher flow speed (0.8 mm/s) compared to biofilms under lower flow velocity (0.2 mm/s) of the media (Schroll et al., 2010). Temperature, pH, the availability of nutrients, and substrate composition all directly affect the formation of bacterial biofilms (Ribeiro et al., 2016). Further investigation into the mechanisms of biofilm formation in *K. pneumoniae* will ultimately facilitate the treatment of biofilm-related infections, thus reducing mortality and morbidity in patients with life-threatening infections.

## 3 Biofilm and its association with antibiotic resistance

The global spread of antibiotic-resistant strains of *K. pneumoniae* has become a critical concern; particularly in light of the increasing prevalence of broad-spectrum β-lactamases (ESBLs) and carbapenase-producing strains. Biofilm bacteria, relative to planktonic bacteria, enhances the antibiotic resistance to several antibiotics including ampicillin, ciprofloxacin, gentamicin, and cefotaxime (Anderl et al., 2000). Correlation between biofilm formation and infection sites showed varying results in different studies. Shadkam et al. analyzed the antibiotic resistance and biofilm formation of 100 non-duplicative *K. pneumoniae* collected from urine, wound exudates, intratracheal tube (ITT), blood, and sputum. The results indicated that biofilm-producers accounted for 75% of *K. pneumoniae* where biofilm formation in MDR isolates was significantly higher than in non-MDR isolates (P < 0.05), the biofilm-formation ability of the sputum isolates was significantly higher compared to other isolates (P < 0.001) (Shadkam et al., 2021). Ashwath et al. found that 97.1% of the clinically multidrug-resistant (MDR) *K. pneumoniae* isolates formed biofilms, and the isolates from blood, pus and trachea secretions were more capable of forming biofilms (Ashwath et al., 2022). Tuncer et al. investigated biofilm-forming features of pan-resistant (resistant to all agents) *K. pneumoniae* and found that all the isolates formed high-level biofilms, whereas no significant difference was detected for the isolates obtained from different samples (blood, sputum and wound) (Tuncer et al., 2022). Differences between different studies may attribute to geographic regions, sample types and numbers, and bacterial antibiotic resistance.

The biofilm formation intensities of drug-resistant strains have significant correlation with antibiotic resistance and biofilm formation. Analysis of biofilm formation among 137 K*. pneumoniae* strains from sputum and urine revealed that 85.0% (51/60) of biofilm-positive strains had the ability to produce extended-spectrum beta-lactamases (ESBLs) compared to biofilm negative strains with a rate of 11.7% (9/77) (Yang and Zhang, 2008). Subramanian et al. reported that in 100 urine isolates, the resistance rates to ampicillin and cefotaxime were 83.3% and 73.3% in biofilm forming isolates, respectively, and were only 60% and 35% for non-biofilm-forming isolates, respectively (Subramanian et al., 2012). Rahdar et al. found that the carbapenem resistance phenotype significantly correlated with the biofilm formation ability of *K. pneumoniae*, and 99.9% of carbapenem resistant isolates formed medium and high strength biofilms (Rahdar et al., 2019). Extensively drug-resistant (XDR) *K. pneumoniae* isolates also showed greater ability to form biofilm (91.07%) when compared to MDR and sensitive strains, indicating a positive correlation between antibiotic resistance profile and biofilm-forming ability. A significant relationship between strong biofilm formation and prevalence of VIM (Verona integron-encoded metallo-β-lactamase) and IMP (Imipenemase) genes was reported (Khodadadian et al., 2018).

Biofilm can also form in antibiotic sensitive isolates, and resistant strains can be non-biofilm producers. Zheng et al. analyzed biofilm formation in 250 K*. pneumoniae* bacteremia isolates, but no significant association was found between biofilm formation and resistance to the examined antibiotics (Zheng et al., 2018). Cusumano et al. investigated 139 clinically isolated *K. pneumoniae* strains and found that multi-drug resistant isolates (n=81) more commonly formed weak biofilms, and XDR (n=25) isolates were similar between the groups (Cusumano et al., 2019). Carbapenem-resistant *K. pneumoniae* (CRKP) were less likely to form a strong biofilm. Fang et al. also reported that CRKP was more likely to form weak biofilms compared to carbapenem-sensitive strains by analysis of 40 imipenem-resistant strains and 40 imipenem-sensitive strains (Fang et al., 2021). Strong biofilm formation in carbapenem-sensitive strains maybe caused by the *mrkH* gene, which is more widely distributed in carbapenem-sensitive strains than in carbapenem-resistant strains. Sabenca et al. evaluated biofilm formation of KPC-Producing and ESBL-Producing *K. pneumoniae*, and found that most ESBL- and KPC-producing isolates were weak biofilm producers (40.0% and 60.0%). The presence of ESBL and KPC enzymes was not related with the ability to form stronger biofilms (Sabença et al., 2023). Hence, the correlation between antibiotic resistance and biofilm formation awaits further research.

**Figure 3:**
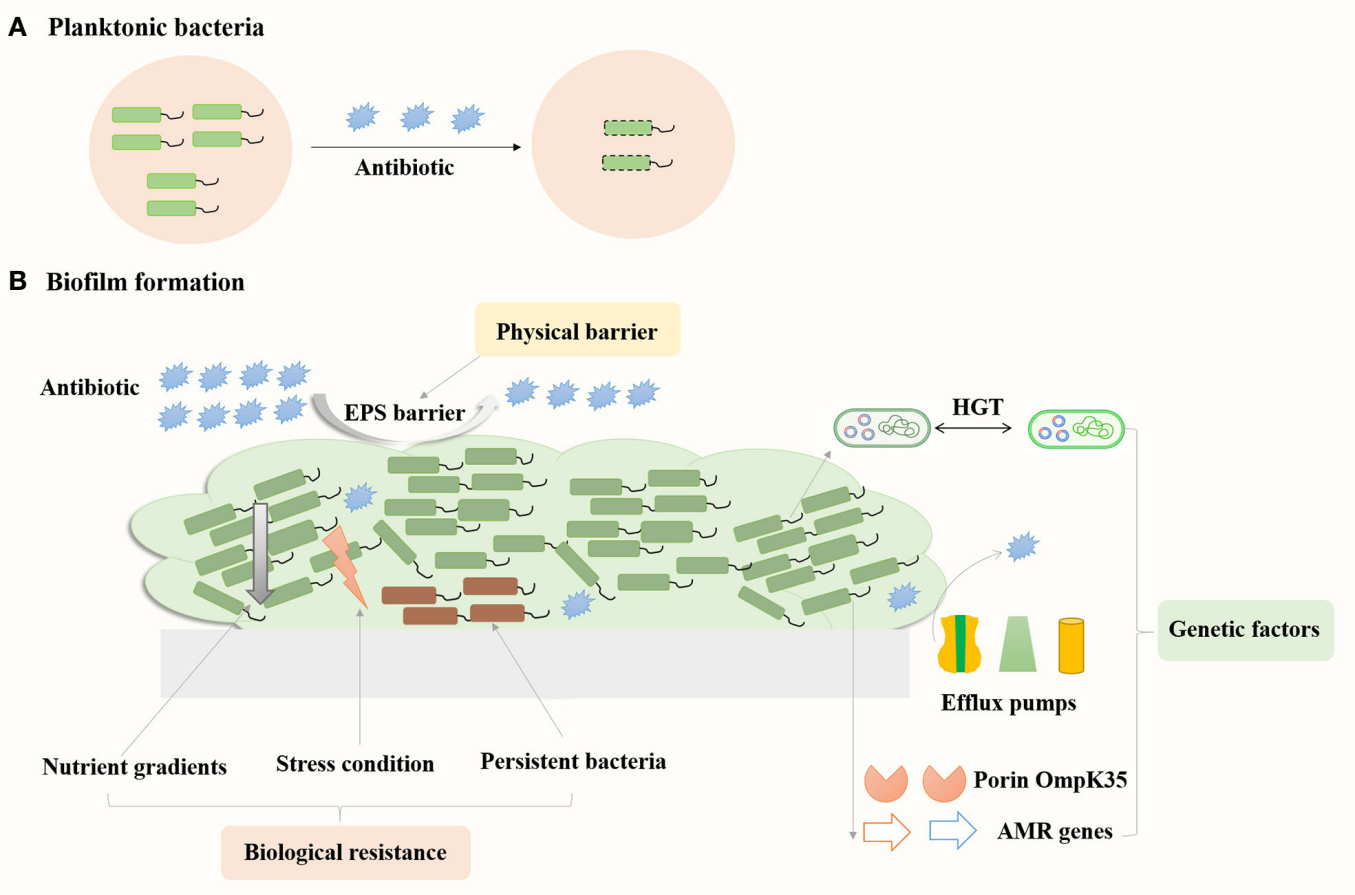
Mechanisms contribute to biofilms related resistance in *K. pneumoniae*. Bacteria in the floating state can be killed by antibiotics **(A)**, while bacteria in the biofilm state are more resistant to antibiotics **(B)**.

Biofilm resistance mechanisms mainly consist of three categories: physical barrier, biological resistance, and genetic factors (**Figure 3**). The physical barrier is mainly through the barrier protection by the EPS, which can prevent antibiotics from penetrating the biofilm and reaching the bacterial cells, resulting in increased antibiotic resistance (Wu et al., 2011). Desai et al. reported increased amounts of eDNA, protein and exopolysaccharides (EPS) in the strong biofilms of *K. pneumoniae* compared to weak biofilm producers (Desai et al., 2019). Meanwhile, levofloxacin treatment could induce the production of EPS (exoproteins and exopolysaccharides) in *K. pneumoniae* biofilms and prevent the spread of levofloxacin, thus increasing the bacterial resistance to levofloxacin (Zhang et al., 2021). Biological resistance mainly consists of persistent bacteria, nutrient gradients and stress responses. Persistent bacteria are subgroups of bacteria that enter a dormant state, exhibiting reduced metabolic activity and increased tolerance to antibiotics, which can survive antibiotic treatments and resume growth, contributing to recurrent infections and chronic disease states. The antibiotic susceptibility of planktonic bacteria substantially reduced when they enter the stationary phase under nutrient limitation conditions (Anderl et al., 2003). In the early stage of biofilm development, the specific growth rate of bacteria in the colony was 0.49 h^−1^, similar to that of planktonic bacteria of 0.59 h^−1^, whereas the growth rate was 0.032 h^−1^ as the colony matured (Anderl et al., 2003). Zhang et al. reported that levofloxacin treatment could eliminate *K. pneumoniae* in the planktonic state, whereas resulted in thicker biofilm formation and the persistent state of the bacteria in the biofilms (Zhang et al., 2021). *K. pneumoniae* on the biofilm could enter into the suspension for secondary growth under suitable conditions. In addition, Li et al. reported that low concentrations of antibiotics could induce the formation of persister cells, and *K. pneumoniae* produces high levels of tolerant persister cells to survive high concentrations of bactericidal antibiotics (Li et al., 2018). Amino acid starvation could increase the biofilm survival percent of *K. pneumoniae* strains treated with ceftazidime from 22–41% to 70 to 388% (Davis and Brown, 2020). Thus, the bacteria in the persistent state could tolerate the nutrient limitation and stress conditions in the biofilms and increase bacterial antibiotic resistance. Major genetic factors include exogenous DNA and horizontal gene transfer between bacteria, which led to the changes in expression of specific genes in biofilms. Increased expression of multidrug efflux pumps could actively extrude antibiotics from bacterial cells, reducing antibiotic concentrations (Kvist et al., 2008). Relative transcription levels of genes encoding efflux pump increased in *K. pneumoniae* biofilms compared to the planktonic cells (Tang et al., 2020). Meanwhile, addition of the efflux pump inhibitor such as thioridazine, Phe-Arg β-naphthylamide (PAβN) and NMP significantly reduced biofilm formation of *K. pneumoniae*. The presence of NMP increased the sensitivity of overnight biofilms of *K. pneumoniae* to tetracycline (Kvist et al., 2008). Compared to planktonic cells, the gene encoding porin OmpK35 and the *acrB* gene encoding multidrug-efflux pump upregulated in XDR *K. pneumoniae* biofilms (Vuotto et al., 2017). Therefore, understanding these mechanisms is crucial for developing effective strategies to tackle biofilm-associated antibiotic resistance in *K. pneumoniae* infections.

## 4 Novel approaches for treatments of biofilm-related infections

### 4.1 Drug combination

**Figure 4:**
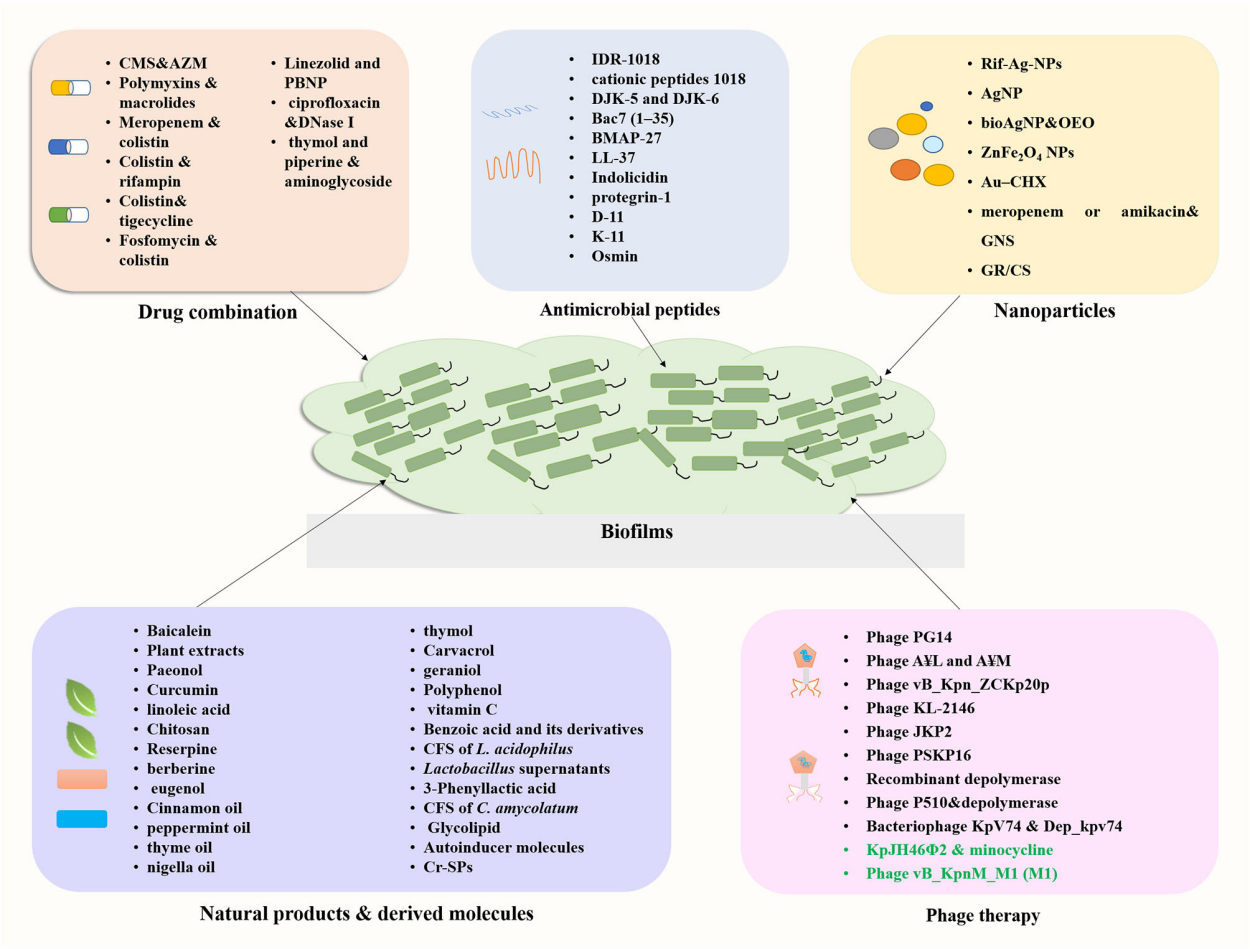
Summary of novel approaches for the treatment of biofilm related infections.

Emergence of infections caused by carbapenem-resistant *K. pneumoniae* (CRKP), especially that involving biofilm formation, is associated with high rates of morbidity and mortality. The therapeutic options available are currently limited. The limited availability of new antibiotics in the drug development pipeline has prompted a revival in the use of old antimicrobials and researches into drug combinations. This section summarizes recent research focusing on the effectiveness of drug combinations in eradicating *K. pneumoniae* biofilms (**Figure 4**; **Supplementary Table S1**).

#### 4.1.1 Combination of antibiotics

Certain less potent antibiotics, like macrolides, can be effective, when applied in combination with antibiotics that have a different mechanism of action. Polymyxins are a group of polypeptide antibiotics, mainly consisting of polymyxins B and E (colistin), which are known for their ability to inhibit the formation of biofilms and to reduce pre-existing biofilms (Herrera et al., 2019). Colistin sulfate (COL) and colistin methanesulfonate (CMS) are improved versions of colistin. Combination of CMS and azithromycin (AZM) showed a synergistic antibacterial effect against both planktonic growth and biofilm formation in *K. pneumoniae* (Moshynets et al., 2022). She et al. demonstrated synergistic antibacterial and antibiofilm activity between a polymyxin B derivative SPR741 and macrolide antibiotics (erythromycin and clarithromycin) against extensively drug-resistant and pandrug-resistant *K. pneumoniae* (She et al., 2022). SPR741 in combination with macrolide antibiotics (double or triple combination) could effectively eradicate highly resistant bacterial biofilms and persistent cells *in vitro* and *in vivo*, indicated by laser confocal microscopy and colony forming unit-counting results (She et al., 2022). The combination of 8 µg/mL SPR741, 16 µg/mL clarithromycin, and 16 µg/mL erythromycin was found to be the most potent against pre-formed biofilms (48 hours). Moshynets et al. investigated the therapeutic potential of AZM-CMS combination against XDR *K. pneumoniae* isolates using 3D Collagen-Based *in vitro* wound model of biofilm infection, and the results confirmed AZM to be an effective antibiofilm drug used either alone or in combination with CMS (Moshynets et al., 2023).

Other antibiotics may also have increased antibiofilm efficacy used in combination with polymyxins. Ribera et al. conducted a pharmacodynamic model mimicking biofilm formation to compare the efficacies of meropenem alone and in combination with colistin against extended-spectrum-β-lactamase-producing *K. pneumoniae* (Ribera et al., 2019). They found that the combination of meropenem monotherapy and colistin-meropenem exhibited comparable efficacy, but the drug pairing could also prevent the emergence of colistin-resistant subpopulations. Geladari et al. investigated the effects of colistin (CST), rifampin (RIF), meropenem (MEM), gentamicin (GEN), and tigecycline (TGC) both individually and in combination with CST against mature biofilms of CRKP (Geladari et al., 2019). Combinations of CST (32-64 mg/L) and RIF (0.25-4 mg/L), CST (32 mg/L) and MEM (0.007-0.25 mg/L), and CST (16-32 mg/L) and TGC (16-64 mg/L) exhibited a synergistic effect where the highest synergistic effect was observed for CST and RIF. Synergistic activity of fosfomycin/colistin combined against biofilms of Gram-negative strains including *K. pneumoniae* were also reported where the synergistic effects were not species-related or dependent on their MICs, resistance mechanisms, or clonal lineage (Boncompagni et al., 2022). The synergistic antibiofilm activity of fosfomycin in combination with polymyxin B or meropenem against KPC-2-producing *K. pneumoniae* clinical isolates (KPC-KPN) were reported (Ribeiro et al., 2023). Biofilm disruption was observed when exposed to antimicrobials alone and in combination. Fosfomycin in combination with polymyxin B resulted in a 2.4–3.4 fold reduction in biofilm formation; whereas fosfomycin alone and polymyxin B alone reduced formation by 2.231–3.470 and 2.378–3.423 fold, respectively. For the combination of fosfomycin and meropenem, as well as meropenem alone, biofilm formation decreased by 1.481–2.724 fold and 1.335–2.385-fold, respectively, with two isolates reducing to a non-adherent state (Ribeiro et al., 2023). Higher biofilm disruption was observed for fosfomycin in combination with polymyxin B, followed by polymyxin B and fosfomycin alone (p < 0.001). Linezolid showed potential in the development of combination therapeutic agents for its synergistic antibacterial activity against multidrug-resistant pathogens. Huang et al. reported that combination of linezolid and polymyxin B nonapeptide PBNP (LP) significantly reduced the biofilm production of *K. pneumoniae* and exhibited significant protection against *K. pneumoniae* infection in *Caenorhabditis elegans* (Huang et al., 2022).

#### 4.1.2 Combination of antibiotics with other antibiofilm components

Combination of antibiotics with other antibiofilm components could boost their effectiveness in inhibiting biofilm formation. Sharma et al. reported that the use of DNase I in conjunction with ciprofloxacin resulted in an 8-fold and 4-fold increase in the biofilm-eradicating ability of the antibiotic in *K. pneumoniae* ATCC 700603 and a clinical isolate, respectively, and achieved a 99% reduction of biofilm biomass in a mouse model (Sharma et al., 2023). Ndezo et al. reported the synergistic antibiofilm activity of thymol and piperine when combined with three aminoglycoside antibiotics against *K. pneumoniae* biofilms, with the minimum biofilm eradication concentration reduced 16- to 64-fold for the combination of thymol and streptomycin or kanamycin, and 8- to 16-fold for the combination of piperine and kanamycin (Bisso Ndezo et al., 2021).

In summary, combining traditional antimicrobials with antibiotics that have diverse action mechanisms or incorporating other anti-biofilm agents may result in unforeseen effectiveness against biofilms caused by *K. pneumoniae*. Further *in vivo* research is necessary to facilitate the clinical use of drug combinations and enhance the efficacy of anti-infections.

### 4.2 Antimicrobial peptides

Antimicrobial peptides (AMPs) are small proteins composed of 10–50 amino acids present in different organisms, which are effective against a range of pathogens including bacteria, viruses, fungi, and parasites (de Souza et al., 2023). AMPs are promising novel therapeutic agents for their broad-spectrum antibiotic activities and special mechanisms of action. In addition, AMPs can have biofilm inhibition activity either used alone or in combination with antibiotics, making them candidates for treating biofilm-associated infections (Zhao et al., 2023). Here, we summarize recent progress on the study of AMPs with antibiofilm activity against *K. pneumoniae* (**Figure 4**; **Supplementary Table S1**).

Immunomodulatory peptide IDR (innate defense regulator)-1018 is a broad-spectrum antibiofilm peptide with activity against multiple antibiotic-resistant species, which functions by affecting stringent response mediated by (p) ppGpp (de la Fuente-Núñez et al., 2014). IDR-1018 has been shown to prevent biofilm development and reduce existing biofilms by inhibiting (p) ppGpp accumulation in many pathogens including *K. pneumoniae* (de la Fuente-Núñez et al., 2014). Synthetic cationic peptides 1018 (a variant of host defense peptides) and DJK-5 and DJK-6 (D-enantiomeric protease-resistant peptides) were reported to have antimicrobial and antibiofilm activity for carbapenemase-producing *K. pneumoniae* (Ribeiro et al., 2015). The application of peptides 1018 and DJK-6 on mature biofilms of KpC isolates led to disruption of two-day-old biofilms, resulting in the dispersal of biofilm into monolayer cells or single cells, as observed in flow cell experiments. Moreover, peptide DJK-6 was found to enhance the capacity of meropenem (16-fold) in eradicating preformed biofilms. Sub-inhibitory concentrations of Bac7 (1–35) or BMAP-27 caused about a 40% decrease in biofilm formation of *K. pneumoniae* clinical isolates (Benincasa et al., 2016). Confocal microscopy analysis indicated different effects of high concentration AMPs on matured biofilm architecture. BMAP-27 treatment led to a reduction of biofilm height by 36% ± 6%, and the reduction of total biomass by 75% ± 0.2%, resulting in a scattered biofilm structure. Treatment with Bac7 (1–35), on the other hand, reduced the biofilm height by 21% ± 14%, whereas the biomass was not affected, and the biofilm structure was denser than the control. Four host defense peptides namely LL-37, indolicidin, protegrin-1 and bac7 (1-35) have been shown to exhibit antimicrobial activity towards *K. pneumoniae*, which aggregates with capsule polysaccharides to exert their effect (Fleeman and Davies, 2022). Polyproline peptide bac7 (1-35) and protegrin-1 exhibited superior activity in eliminating preformed biofilms of hypermucoviscous strains compared to non-hypermucoviscous strains. Bac7 (1-35) demonstrates the highest efficacy in eradicating biofilms among the tested strains, particularly for hypermucoviscous strains like *K. pneumoniae* NTUH K2044. The human cathelicidin-derived peptide D-11 had synergy with 13 antibiotics, mainly from the families of aminocoumarins, macrolides and rifamycins, which were tested using a bacteremia model and mouse abscess model for *K. pneumoniae* infections (Cebrián et al., 2021). K11 (KWKSFIKKLTKKFLHSAKKF-NH2) is a novel antimicrobial peptide derived from natural AMPs (cecropin A1, melittin, and maganin 2), which was reported to have antimicrobial and antibiofilm activity against MDR and extensively drug-resistant (XDR) *K. pneumoniae* (Chatupheeraphat et al., 2023). K11 displayed a dose-dependent biofilm inhibition against four strong-biofilm-forming strains of MDR/XDR *K. pneumoniae*, causing approximately 32% to 80% decrease in the biofilm biomass at the concentration of 0.25 × MIC to 1 × MIC. Synergistic effects were observed for K11 combined with chloramphenicol, meropenem, rifampicin, or ceftazidime, whereas no synergy was observed with colistin. Osmin is a well-known bee venom peptide composed of 17 amino acids (GFLSALKKYLPIVLKHV-NH_2_), which exhibited significant antibiofilm activity against the *K. pneumoniae* KCTC 2208 and CRKP strains (Artini et al., 2023; Jeon et al., 2023). Osmin inhibited 93.3% (at 1.56µM) biofilm formation of *K. pneumoniae* KCTC 2208, and more than 85% (at 3.13 µM) biofilm formation of CRKP strains. Osmin at 100 µM could eradicate over 60% preformed biofilm and the rate of was 80.5% for *K. pneumoniae* KCTC 2208 and 62.5-90.1% for CRKP strains. A CRKP-septic mouse model was constructed to analyze the therapeutic effect of osmin, and the results indicated that osmin could reduce the bacterial load in the blood and organs of the mice, reduce pro-inflammatory cytokine expression, and ameliorate tissue damage. After 30 generations in osmin-containing medium, CRKP strains did not develop drug resistance to Osmin, making it a promising treatment for drug-resistant *K. pneumoniae* infections.

In summary, AMPs are promising for preventing biofilm formation and disrupting preformed biofilms for both *K. pneumoniae* ATCC strains and clinical isolates. Due to their different mechanisms of action, these AMPs may work through affecting biofilm matrix components, which can be utilized in combination with other antibiotics to enhance the antibiofilm efficacy. Hence, AMPs are promising candidates for treating multidrug resistance, pending further research.

### 4.3 Nanoparticles

Nanoparticles (NPs) such as metallic nanoparticles, liposomes, and dendrimers are attracting increasing interests due to the lack of antibiotics and emerging drug resistance (Ferreira et al., 2021). NPs are considered as effective antimicrobial agents due to their large surface area and ease for surface modification. The main mechanisms of action of nanoparticles included cell membrane alterations and disruption, ROS generation, lipid peroxidation and metabolic pathway disruption (Sánchez-López et al., 2020; Cruz et al., 2021). Here, we summarize recent progress on the study of nanoparticles with antibiofilm activity against *K. pneumoniae* (**Figure 4**; **Supplementary Table S1**).

Metal nanoparticles have been studied as a potential alternative to combat bacterial antibiotic resistance, specifically for anti-biofilm infections caused by *K. pneumoniae*. Farooq et al. synthesized rifampicin conjugated silver nanoparticles (Rif-Ag-NPs), which was found to inhibit > 90% *K. pneumoniae* biofilm formation at low doses compared to rifamipicin alone (Farooq et al., 2019). Additionally, Rif-Ag-NPs showed 1.5-2-times biofilm eradication activity compared to rifampicin alone. Siddique et al. reported the efficacy of silver nanoparticle AgNP in inhibiting biofilm formation by reducing the production of extracellular polymeric substances, and AgNPs (100 μg/ml) exhibited a percentage inhibition of 64% for *K. pneumoniae* strain MF953600 and 86% for MF953599 (Siddique et al., 2020). In another study, Scandorieiro et al. demonstrated that the combination of oregano essential oil (OEO) and their bioactive components with biogenic silver nanoparticles (bioAgNP) enhanced bacteriolytic activity (Scandorieiro et al., 2023). Oregano essential oil (OEO), carvacrol (Car) and thymol (Thy) alone have been shown to prevent biofilm formation and decrease metabolic activity of preformed biofilm in KPC-producing *K. pneumoniae*. When used in combination, all three were found to have improved anti-biofilm activity compared to antimicrobials used individually. The combination of Thy and bioAgNP was particularly effective in inhibiting biofilm formation and disrupting the biofilm structure formed on polystyrene and glass surfaces.

Additionally, Zinc ferrite nanoparticles (ZnFe_2_O_4_ NPs) have shown excellent antibacterial efficiency against *K. pneumoniae* by inducing reactive oxygen species (ROS)-induced bacterial damage and disrupting bacterial membrane integrity (Sharma et al., 2022). ZnFe_2_O_4_ NPs were found to inhibit biofilm formation up to 81.76% and reduce mature biofilm up to 56.22% at 75 μg/mL. Ahmed et al. reported antibiofilm efficacy of gold nanoparticles conjugated with chlorhexidine (Au–CHX) against *K. pneumoniae* isolates (Ahmed et al., 2016). Au-CHX was effective both in inhibiting early-stage biofilm formation and in eradicating established biofilms. The treatment significantly reduced the level of *K. pneumoniae* biofilm by 85%-90%. Aguilera-Correa et al. evaluated the antibacterial effect of gold nanostars (GNS) alone, and in combination with meropenem or amikacin against both planktonic and biofilm form CRKP strains (Aguilera-Correa et al., 2022). The combination of 4 µg/mL amikacin with GNS concentrations greater than 80 µM was found to inhibit biofilm growth of *K. pneumoniae* strains, whereas inhibitory effects on ATCC23357 biofilm were observed when combined with 2µg/mL meropenem and various concentrations of GNS. Therefore, NPs used independently or in combination with other antibiofilm agents show promise in the development of novel anti-biofilm drugs.

Graphene (GR) and graphene/chitosan nanoparticles (GR/CS NCs) exhibited antibiofilm activity against *K. pneumoniae* (Muthuchamy et al., 2020). Treatment with 70μg/mL of GR led to 90% reduction of biofilm production by *K. pneumoniae*, whereas treatment with 40μg/mL of GR/CS NCs resulted in a 92% reduction in biofilm formation. These results suggest that GR/CS NCs were more effective in inhibiting biofilm formation than GR alone. In summary, nanoparticles metal-based or dendrimers are also promising candidates in the antibiofilm against *K. pneumoniae*, which can be used alone or with other antimicrobial drugs.

### 4.4 Natural products derived molecules

Natural products or derivatives with fewer side effects have been sought for the control of MDR infections and inhibition of bacterial biofilm. These products mainly include plant derived molecules such as Chinese medicine, plant extracts, essential oils traditional tea, and microbials and their metabolites such as probiotic strains. Various research groups have investigated the application of natural agents against *K. pneumoniae* biofilms. Here, we summarize recent progress on the study of natural products or their derived molecules with anti-biofilm activity against *K. pneumoniae* (**Figure 4**).

#### 4.4.1 Plant derived molecules

Traditional Chinese medicine has become a rich resource for the discovery of alternative synergistic antibacterial agents. Baicalein is a type of flavonoid isolated from the roots of *Scutellaria baicalensis* and *Scutellaria lateriflora*, which has been used in China for the treatment of bacterial infections. Analysis of baicalein with cefotaxime revealed that baicalein exhibited synergistic effects on some antibiotic-resistant ESBL-positive strains of *K. pneumoniae* (56.3%) and the positive control clavulanate acid showed 100% synergy (Cai et al., 2016). Plant extracts from *Pfaffia paniculata* K. (55.6%) and *Rosmarinus officinalis* L. (58.1%) significantly reduced planktonic, and biofilm formation by *K. pneumoniae*, showing its potential for the treatment of bacterial infections (Paula-Ramos et al., 2016). Paeonol, which is primarily extracted from peonies and the root bark of *Paeonia suffruticosa*, is a phenolic compound with a wide range of pharmacological effects and has been applied in China for half a century for inflammation/pain-related indications (Zhang et al., 2019). Paeonol has demonstrated effective antibacterial and anti-biofilm activities against *K. pneumoniae* both in the planktonic and biofilm states at concentration of 64 μg/mL by disrupting the integrity of bacterial cell membranes and altering cell morphology (Qian et al., 2021).

Plant-derived natural compounds have been demonstrated with significant anti-biofilm properties. Six natural compounds (curcumin, eugenol, linoleic acid, chitosan, reserpine and berberine) were tested for their inhibitory effects on *K. pneumoniae* strain growth and biofilm formation, and the data indicated that 6 natural compounds could inhibit biofilm formation in high biofilm forming isolates (Magesh et al., 2013). Among them, reserpine was the most potent biofilm inhibitors followed by linoleic acid. Reserpine was an efflux pump inhibitor and linoleic acid was an essential fatty acid. Mohamed et al. showed that essential oil alone or in combination with ciprofloxacin could inhibit/eradicate biofilms in MDR *K. pneumoniae*, suggesting the potential of essential oil against related infections (Mohamed et al., 2018). Seven essential oils were analyzed including caraway oil, cinnamon oil, clove oil, ginger oil, nigella oil, peppermint oil and thyme oil. Cinnamon oil had the best antibacterial activity against planktonic cells followed by clove oil, whereas no observable antibacterial activity could be detected for ginger, nigella and peppermint oils (Mohamed et al., 2018). Surprisingly, peppermint oil showed significant biofilm inhibiting ability with a 98.2% inhibition percentage compared to 95.2% for thyme oil and 77.2% for nigella oil. Thyme oil had the best biofilm eradication ability with a eradication percentage of 80.1 to 98.0%, followed by peppermint and cinnamon oils (Mohamed et al., 2018). Kwiatkowski et al. investigated the antibiofilm properties of fifteen essential oil compounds (EOCs) against *K. pneumoniae* strains, and found that thymol, carvacrol and geraniol exhibited the most promising antibacterial and antibiofilm activity against uropathogenic New Delhi Metallo-beta-Lactamase-1 (NDM-1) producing *K. pneumoniae* isolates (Kwiatkowski et al., 2022). Eugenol is an important bioactive phytochemical in essential oils of clove. Qian et al. demonstrated that eugenol could inhibit biofilm formation and inactivate biofilm cells in CRKP through damaging of the cell membrane and cell structure (Qian et al., 2020). Liu et al. also reported that eugenol treatment significantly decreased the surface coverage and thickness of CRKP biofilm via the generation of reactive oxygen species (ROS) and the reduction of glutathione (Liu et al., 2023). 1, 8-cineole (also known as eugenol) was shown to have the capability to disrupt biofilm structure and kill cells within the biofilm formed by the multidrug-resistant *K. pneumoniae* strains producing extended-spectrum β-lactamases (Vazquez et al., 2022).

Other plant sources with antimicrobial components have also been reported. Polyphenols are major bioactive components of the traditional beverage tea in China and the effects of tea polyphenols (TPs) on quorum sensing and virulence factors of *K. pneumoniae* were reported (Liu et al., 2020). TPs were shown to decrease production of total proteases and exopolysaccharides of *K. pneumoniae* and TPs at sub-MIC concentrations induced a decrease in biofilm formation in a dose-dependent manner (23.7% inhibition at 200 μg/mL and 44.4% inhibition at 600 μg/mL). Xu et al. demonstrated that vitamin C show a dose-dependent capability to inhibit CR-hvKP growth and the biofilm formation both *in vitro* and in the mouse infection model, which could be attributed to induction of reactive oxygen species (ROS) generation, inhibition of exopolysaccharide (EPS) production and efflux pump (Xu et al., 2022). Benzoic acid and its derivatives are promising antimicrobial candidates, among which 3-hydroxy benzoic acid and 2, 5-dihydroxy benzoic acid were reported to inhibit 89-97% of biofilm formation of *K. pneumoniae* (Rohatgi and Gupta, 2023). The treatment of the compounds resulted in the loss of ability for the bacteria to attach to the coverslip surface, and thus the inability to form biofilms. These findings suggest that plant-derived compounds could be potential sources of new drugs to fight biofilm-associated infections.

#### 4.4.2 Microbial and their metabolites

Microorganisms and their metabolites are also important sources of anti-biofilm drugs (**Figure 4**; **Supplementary Table S1**). *Lactobacillus acidophilus* was the most predominant isolate in yogurt samples, and its cell-free supernatant (CFS) showed inhibitory activity against biofilms of extended-spectrum β-lactamase (ESBL)-producing *K. pneumoniae* (El-Mokhtar et al., 2020). The CFS showed a dose-dependent antibiofilm formation activity against the fresh biofilms of *K pneumoniae*. In addition, 52% ± 12 of the formed biofilms were destroyed when 24 h biofilms were treated with CFS, although the action mechanism was not analyzed. Antibacterial effects on biofilm growth of ESBLs-producing *K. pneumoniae* strains were analyzed for five probiotic *Lactobacillus* strains including *L. rhamnosus* ATCC 7469, *L. acidophilus* ATCC 4356, *L. plantarum* ATCC 8014, *L. casei* ATCC 39392 and *L. fermentum* ATCC 9338 (Kheiri et al., 2020). *Lactobacillus* supernatants exhibited 95% biofilm-inhibitory and biofilm-killing properties against a strong biofilm producing *K. pneumoniae* isolate. The biofilm-killing effect of *Lactobacillus* supernatants were superior to their biofilm-eradicating capacity. Scanning electron microscopy (SEM) analysis revealed that treatment with *Lactobacillus* supernatant resulted in the destruction of the biofilm structure. The antibacterial effect of *Lactobacillus* supernatant was mainly related to its acidic pH and high concentration of hydrogen peroxide. Yu et al. reported that 3-Phenyllactic acid (PLA) produced by lactic acid bacteria could inhibit bacterial growth and biofilm formation of *K. pneumoniae* CVCC4080 (Yu et al., 2023). Meanwhile, PLA could significantly increase the survival rate and reduce the histopathological injury of infected mice.

The probiotic *Corynebacterium amycolatum* isolated from vaginal contents of healthy women also showed antibiofilm activity against imipenem resistant *K. pneumoniae* (Gladysheva and Cherkasov, 2023). The cell−free supernatants of clinical isolated *C. amycolatum* strains could reduce biofilm formation of *K. pneumoniae* with the inhibition rate ranged from 3.95% to 39.69%. Meanwhile, the CFS of *C. amycolatum* could destroy preformed biofilms of *K. pneumoniae* with a rate of 7.62% to 19.32%. The antibiofilm activity of *C. amycolatum* CFS was mainly exerted by decreasing autoaggregation, cell surface hydrophobicity and EPS production, thus destroying the structure of biofilms.

Additionally, microbial derived products also showed antibiofilm activity. The glycolipid biosurfactant produced by marine isolate *Shewanella algae* B12 disrupted 87% of the preformed biofilms of *K. pneumoniae* (Gharaei et al., 2022). The biosurfactant may eradicate the biofilm by reducing surface tension and preventing the bacteria from attaching to the surface. Autoinducer molecules known as acylhomoserinelactones (AHL), which are involved in quorum sensing (QS), also showed potential to control biofilm formation. Cadavid et al. tested twenty-seven compounds structurally similar to QS inhibitors, and found that 3-methyl-2(5H)-furanone and 20-hydroxycinnamic acid inhibited biofilm formation by 67.38% and 65.06%, respectively (Cadavid and Echeverri, 2019). The inhibition mechanism of 3-methyl-2(5H)-furanone was analyzed by adding the compounds at different stages of biofilm formation, and the results indicated that it affected the adhesion of the strain, thus decreasing the formation of mature biofilm. Meanwhile, the compound could change biofilm structure and the proportion of different sized bacteria in the biofilm. The sulphated polysaccharides produced by green algae *Chlamydomonas reinhardtii* (Cr-SPs) also showed antibacterial and antibiofilm potential against *K. pneumoniae* (MTCC no. 432) (Vishwakarma et al., 2022). Cr-SPs treatment eradicated more than 50% of preformed biofilm at 0.5 mg/mL and removed 100% biofilm at 4 mg/mL and 8 mg/mL by reducing EPS production and eDNA content of the bacteria.

In summary, various natural products or their bioactive components have been shown to have antibacterial effects on biofilm growth of *K. pneumoniae*, thus making them a novel therapeutic approach for biofilm related infections. Further research should focus on conducting *in vivo* and clinical trials for the promising candidates.

### 4.5 Phage therapy

Bacteriophages (phage) are viruses that infect and kill bacteria with the advantages of high functional specificity, tolerance, safety, narrow range of action and cost-effectiveness (Principi et al., 2019). Phage therapy was recognized as a promising alternative therapy for bacterial infections for the following reasons: (i) different resistance mechanism from that of antibiotics, (ii) only a few side effects, and (iii) higher penetrating capacity to destroy biofilms (Shariati et al., 2022). An increasing number of researchers have focused on phage therapy for a variety of the ESKAPE pathogens, which provides evidence for lytic phage as an alternative to antibiotics (da Rosa et al., 2020; Nazir et al., 2022). Several lytic phages have shown the potential to combat MDR *K. pneumoniae* infections through *in vitro* or *in vivo* studies, as reviewed by Herridge et al. (2020). On the other hand, we aim to discuss the ability of phages to combat bacteria in the biofilm state (**Figure 4**; **Supplementary Table S1**).

#### 4.5.1 Phages in the research stage

Effective antibiofilm phages for MDR *K. pneumoniae* infections have been reported. A *Klebsiella* phage PG14 can lyse carbapenem resistant *K. pneumoniae* G14 and show significant antibiofilm efficacy with 80% biofilm inhibition and 71% biofilm disruption (Mulani et al., 2022). Phage A¥L and A¥M, belonging to *Myoviridae* and *Siphoviridae* family, were reported to have biofilm inhibition activity with an eradication rate of 50-70% against 48 h mature biofilm by killing most bacteria within the biofilms. Compared to the control group, treatment with phage led to a distorted biofilm morphology and bacterium death. Phage vB_Kpn_ZCKp20p isolated from urban and medical sewage showed the ability to lyse biofilm-producing MDR *K. pneumoniae* isolates without cytotoxicity to human skin fibroblasts, and could inhibit biofilm formation and disrupt mature biofilms (Zaki et al., 2023). Polyvalent phage KL-2146 can infect NDM producing *Klebsiella* and antibiotic-sensitive *K. pneumoniae* 13, 883, which was demonstrated to effectively disrupt biofilms possessing multiple *Klebsiella* strains (Gilcrease et al., 2023). Asif et al. isolated a K-17 serotype specific *K. pneumoniae* phage JKP2, which could significantly eliminate preformed biofilms with a rate of 98% for 24-hour-old biofilm, 96% of 48-hour-old biofilm, 86% and 82% for mature biofilm on day 3 and 4, respectively (Asif et al., 2023). Rahimi et al. characterized a lytic phage PSKP16 with therapeutic potential against β-lactamase and biofilm producing K2-Hypervirulent *K. pneumoniae* using a mouse pneumonia model (Rahimi et al., 2023). PSKP16 could reduce 18–64.6% of the 24-hour-old biofilms and 16.4–63.7% of the 48-hour-old biofilms. In the pneumonia model, the effect of the timely administration of phages PSKP16 was faster for reducing bacterial load and improving survival compared to the delayed synergistic model, but the endpoints were fairly similar. Phage treatment exceeded the therapeutic effect of gentamicin alone, and there was no severe lesions and alveolar edema, with reduced inflammatory cell infiltration.

Phages encoded proteins also gained increasing attention for their role in antibiofilm and antibacterial activities. These proteins included endolysins, virion-associated lysins (VALs), polysaccharide depolymerase (Dep) and the receptor binding proteins (RBPs) (Herridge et al., 2020; Anyaegbunam et al., 2022). Recombinant depolymerase 42 (Dep42) of the Phage SH-KP152226 of *K. pneumoniae* capsular type K47 showed antibiofilm activity (Wu et al., 2019). Dep42 showed specific enzymatic depolymerization of *K. pneumoniae* K47 capsule and significantly inhibited biofilm formation and degraded mature biofilms. Furthermore, when used in combination with polymyxin, Dep42 could enhance its activity against *K. pneumoniae* biofilms (Wu et al., 2019). Li et al. isolated a lytic phage P510 from *K. pneumoniae* KL64 and characterized a specific phage-derived depolymerase with polysaccharide-degrading activity and significant antibiofilm effect, which had the same lysis spectrum as phage P510 (Li et al., 2021). Bacteriophage KpV74 and phage depolymerase Dep_kpv74 are specific to lyse hypervirulent *K. pneumoniae* of the K2 capsular type, and the depolymerase Dep_kpv74 was effective against *K. pneumoniae* infection in mice thigh soft tissue, with comparable or greater efficiency than that of the bacteriophage (Pertics et al., 2023).

Phage resistance is also an emerging question that requires further research. Phage 117 was reported to have strong lytic activity towards the host *K. pneumoniae*, but rapid regrowth was observed, whereas a phage cocktail (117 and 31) showed significantly higher antimicrobial activity than phage 117 alone (Tan et al., 2019). Townsend et al. reported that the *Klebsiella*-infecting lytic phages are most suitable for phage therapy, whereas a single phage was not able to suppress the growth of *Klebsiella* for more than 12 h, possibly due to the emergence of spontaneous phage-resistant mutants (Townsend et al., 2021). Li et al. also demonstrated that phage cocktail were more effective in reducing bacterial densities (Li et al., 2021). Three phages (NL_ZS_1, NL_ZS_2, and NL_ZS_3) were isolated from ST11 CRKP isolates, which showed strong lytic potential, but were followed by the rapid emergence of phage-resistant mutants. Zurabov et al. reported the inhibition of multidrug-resistant *K. pneumoniae* Kl 315 biofilms using a cocktail of three bacteriophages (vB_KpnS_FZ10, vB_KpnP_FZ12 and vB_KpnM_FZ14) with depolymerase activity (Zurabov et al., 2023). For the preformed and mature biofilms, the treatment of phage cocktail led to the disruption of biofilms with only single cells and small colonies observed on the glass. The antibiofilm activity of the phage cocktail was similar to that of single vB_KpnP_FZ12. Hence, a cocktail of multiple phages or in combination with antibiotics would be vital for an effective phage therapy for the treatment of *Klebsiella* infections.

#### 4.5.2 Phage therapy used as last resort treatment clinically

Successful cases of phage therapy in clinical use have been reported (Cano et al., 2021; Eskenazi et al., 2022). Intravenous phage therapy of a single phage (KpJH46Φ2) targeting *K. pneumoniae* complex alongside continued minocycline was utilized as a limb-salvaging intervention to treat intractable biofilm-associated prosthetic knee infection for a 62-year-old patient with diabetes. Phage therapy resulted in successfully alleviation of local symptoms and infection indicators without adverse effects with notably biofilm biomass reduction after 22 hours exposure (P = 0.63) (Cano et al., 2021). Eskenazi et al. reported the application of a pre-adapted bacteriophage (vB_KpnM_M1) in combination with antibiotics in the treatment of a fracture-related pandrug-resistant *K. pneumoniae* infection (Eskenazi et al., 2022). The *K. pneumoniae* phage pre-adapted to target the 2 day-170 *K. pneumoniae* isolate was equally active against the day-702 isolate, and was used to treat the isolates from day 702. The salvage treatment improved the clinical, microbiological and radiological symptoms of the patient’s wound and overall condition. *In vitro* analysis showed that the combination of phage vB_KpnM_M1 (M1) and antibiotics had better antibiofilm activity. These results indicated that the combination of phage M1 with antibiotics (meropenem and ceftazidime/avibactam) ultimately leads to the clinical resolution of the patient’s infection. Therefore, there is potential in the application of phage therapy for treating multi-drug resistant bacterial infections. Reports of successful clinical treatment cases suggest that more attempts should be made to use phage therapy clinically, especially as a last resort in cases where there are no treatment alternatives.

## 5 Conclusion and future prospects

*Klebsiella pneumoniae* is a cause of community acquired and hospital acquired infections, and the emergence of multiple drug resistant and biofilm-producing isolates can worsen a patient’s prognosis. Clinically, biofilm formation is associated with 60-80% of bacterial infections, which can protect the pathogen by escaping host immune responses and antibacterial effects of antibiotics, thereby increasing treatment difficulty of related diseases. Hence, it is critical to understand the molecular mechanisms of biofilm formation and its relation with antibiotic resistance in order to provide insight for new drug development and clinical management. *K. pneumoniae* biofilm formation is a process regulated by environmental and genetic factors. Genes related to *K. pneumoniae* biofilm formation mainly include fimbriae, polysaccharides, quorum sensing system and efflux pump. Generally, the biofilm formation intensity of drug-resistant strains may be higher with a significant correlation between antibiotic resistance and biofilm formation, although opposite results also reported. Insufficient availability of effective drugs have resulted in emerging research focusing on novel therapeutic options, including antibiotic combinations, antimicrobial peptides, nanoparticles, natural products or their bioactive components and phage therapy. Although preliminary *in vitro* data of novel drug candidates have been inspiring, further research focusing on the *in vivo* studies and clinical trials for these promising candidates are required to promote the widespread use of these agents.

## Author contributions

LL: Conceptualization, Funding acquisition, Writing – original draft, Writing – review & editing. XG: Conceptualization, Funding acquisition, Writing – review & editing. ML: Data curation, Writing – review & editing. YL: Formal analysis, Writing – review & editing. JM: Formal analysis, Writing – review & editing. XW: Funding acquisition, Writing – review & editing. ZY: Writing – review & editing. WC: Writing – review & editing. WZ: Writing – review & editing. HS: Supervision, Writing – review & editing. XS: Conceptualization, Writing – review & editing. ZW: Conceptualization, Funding acquisition, Writing – review & editing.
