# Supplementary material for: Relationship between biofilm formation and antibiotic resistance of Klebsiella pneumoniae and updates on antibiofilm therapeutic strategies
Source: Front Cell Infect Microbiol. 2024 Feb 23;14:1324895. doi: 10.3389/fcimb.2024.1324895 (PMC10920351; doi:10.3389/fcimb.2024.1324895)
Supplement: Supplementary file 1 [file Table_1.docx]

**Table S1. Summary of novel approaches for treatments of *K. pneumoniae* biofilm-related infections.**

| **Antimicrobial strategies** | **Target strains** | **Antimicrobial susceptibility profiles** | **Experimental models** | **Antibiofilm activity** | **References** |
| --- | --- | --- | --- | --- | --- |
| **Drug combination** | | | | | |
| **Combination of antibiotics** | | | | | |
| Colistin methanesulfonate (CMS) and azithromycin (AZM) | *K. pneumoniae* ATCC 10031 | - | *in vitro* | Synergistic antibacterial effect against both planktonic growth and biofilm formation | [1] |
| Polymyxin B derivative SPR741 and macrolide antibiotics (erythromycin and clarithromycin) | *K. pneumoniae* clinical strains | Extensively drug-resistant and pandrug-resistant | *in vitro* and mice model | Effectively eradicate highly resistant bacterial biofilms and persistent cells *in vitro* and *in vivo* | [2] |
| AZM-CMS | *K. pneumoniae* UHI 1090 | XDR | 3D Collagen-Based *in vitro* wound model | AZM was an effective antibiofilm drug used either alone or in combination with CMS | [3] |
| Meropenem alone and in combination with colistin | Extended-spectrum-β-lactamase-producing *K. pneumoniae* | Resistant to ciprofloxacin but susceptible to meropenem and colistin | *in vitro* | Meropenem monotherapy and colistin-meropenem combination exhibited comparable efficacy, yet the drug pairing can prevent the emergence of colistin-resistant subpopulations. | [4] |
| Rifampin (RIF), meropenem (MEM), gentamicin (GEN), and tigecycline (TGC) individually and in combination with CST | CRKP | Four isolates were resistant to MEM and TGC and sensitive to CST. Three isolates were resistant to GEN, whereas one isolate was GEN sensitive | *in vitro* | The combination of CST (32-64 mg/L) and RIF (0.25-4 mg/L), CST (32 mg/L) and MEM (0.007-0.25 mg/L), and CST (16-32 mg/L) and TGC (16-64 mg/L) exhibited synergistic effect and the highest synergistic effect was observed for CST and RIF combination. | [5] |
| Fosfomycin in combination with polymyxin B or meropenem | KPC-2-producing *K. pneumoniae* clinical isolates | Among the 17 KPC-KPN analyzed, only four were resistant to fosfomycin. Most isolates were susceptible to fosfomycin (82.3%) and resistant to meropenem (88.2%) and polymyxin B (70.6%). All KPC-KPN were resistant to aztreonam, ertapenem, gentamycin, ciprofloxacin, levofloxacin, ceftazidime, ceftriaxone and cefepime. | In vitro and  *Galleria mellonella* model | Higher biofilm disruption was observed for fosfomycin in combination with polymyxin B, followed by polymyxin B and fosfomycin alone. | [6] |
| Linezolid and polymyxin B nonapeptide | *K. pneumoniae* WT strain ATCC 43816 | highly resistant to erythromycin, lincomycin, linezolid, nisin, vancomycin and  polymyxin B derivatives .   susceptible to penicillin G, ampicillin, ciprofloxacin, ofloxacin, tigecycline, and chlortetracycline | In vitro and *Caenorhabditis elegans* | Significantly reduced the biofilm production of *K. pneumoniae* and exhibited significant protection against *K. pneumoniae* infection in *Caenorhabditis elegans.* | [7] |
| **Combination of antibiotics with other antibiofilm components** | | | | | |
| DNase I in conjunction with ciprofloxacin | *K. pneumoniae* ATCC 700603 and a clinical isolate | Susceptible to cefotaxime, ciprofloxacin and amikacin | In vitro and mouse model | 8-fold and 4-fold increase in the biofilm-eradicating ability; a 99% reduction of biofilm biomass in a mouse model. | [8] |
| Thymol and piperine combined with aminoglycoside antibiotics | Four clinical isolates of *K. pneumoniae* | Sensitive or resistant to three aminoglycosides | In vitro | Reduced 16- to 64-fold for the combination of thymol and streptomycin or kanamycin, and 8- to 16-fold for the combination of piperine and kanamycin | [9] |
| **Antimicrobial peptides** | | | | | |
| Immunomodulatory peptide IDR-1018 | *Klebsiella pneumoniae* ATTC 13883 | A colistin-heteroresistant reference strain. | In vitro | Prevent biofilm development and reduce existing biofilms | [10] |
| Cationic peptides 1018 and DJK-5 and DJK-6 | carbapenemase-producing *K. pneumoniae* | Resistant to multiple antibiotics | In vitro | - The application of peptides 1018 and DJK-6 on mature biofilms of KpC isolates led to disruption of two-day-old biofilms. - Peptide DJK-6 enhanced the capacity of meropenem (16-fold) in eradicating preformed biofilms. | [11] |
| Bac7 (1–35) or BMAP-27 | *K. pneumoniae* clinical isolates | - | In vitro | - BMAP-27 treatment lead to a reduction of biofilm height by 36% ± 6% and the reduction of total biomass by 75% ± 0.2%. - Treatment with Bac7 (1–35), on the other hand, reduced the biofilm height by 21% ± 14%, whereas the biomass was not affected, and the biofilm structure was denser than the control. | [12] |
| LL-37, indolicidin, protegrin-1 and bac7 (1-35) | K1 hypermucoviscous strain NTUH K2044, the K2 hypermucoviscous strain 43816, and the nonhypermucoviscous type strain ATCC 13883 | - | In vitro | Polyproline peptide bac7 (1-35) and protegrin-1 exhibit superior activity in eliminating preformed biofilms of hypermucoviscous strains compared to non-hypermucoviscous strains. | [13] |
| Human cathelicidin-derived peptide D-11 | *K. pneumoniae* | - | bacteraemia model and mouse abscess model | D-11 had synergism with 13 antibiotics mainly from the families of aminocoumarins, macrolides and rifamycins. | [14] |
| K11 | Fifteen clinical isolates of *K. pneumoniae* | MDR/XDR | In vitro | K11 displayed a dose-dependent biofilm inhibition causing approximately 32% to 80% decrease in the biofilm biomass. | [15] |
| Osmin | *K. pneumoniae* KCTC 2208 and CRKP strains | Drug resistant | In vitro and septic mouse model | Osmin inhibited 93.3% (at 1.56µM) biofilm formation of *K. pneumoniae* KCTC 2208, and more than 85% (at 3.13 µM) biofilm formation of CRKP strains. | [16] |
| **Nanoparticles** | | | | | |
| Rifampicin conjugated silver nanoparticles (Rif-Ag-NPs) | *Klebsiella pneumoniae* (ATCC: 13882) | - | In vitro | inhibit over 90% *K. pneumoniae* biofilm formation | [17] |
| Silver nanoparticle AgNP | *K. pneumoniae* strain MF953600 and MF953599 | Multidrug-resistant | In vitro | AgNPs (100 μg/ml) exhibited a percentage inhibition of 64% for *K. pneumoniae* strain MF953600 and 86% for MF953599 | [18] |
| Combination of oregano essential oil (OEO) and their bioactive components with biogenic silver nanoparticles (bioAgNP) | KPC-producing *K. pneumoniae* | carbapenem-resistant | In vitro | The combination of Thy and bioAgNP was particularly effective in inhibiting biofilm formation and disrupting the biofilm structure formed on polystyrene and glass surfaces. | [19] |
| Zinc ferrite nanoparticles | *K. pneumoniae MTCC4030* | - | In vitro | inhibit biofilm formation up to 81.76% and reduce mature biofilm up to 56.22% at 75 μg/mL | [20] |
| Gold nanoparticle conjugated with chlorhexidine (Au–CHX) | clinical isolates of *K. pneumoniae* | resistant to  nitrofurantoin, cefuroxime, piperacillin/tazobactam, ceftriaxone  and amoxicillin. | In vitro | Au-CHX was effective both in inhibiting the early stage biofilm formation and in eradicating the established biofilms. The treatment significantly reduced the level of *K. pneumoniae* biofilm to 85%-90%. | [21] |
| Gold nanostars (GNS) alone, and in combination with meropenem or amikacin | CRKP | carbapenem-resistant | In vitro | The combination of 4 µg/mL amikacin with GNS concentrations greater than 80 µM was found to inhibit biofilm growth of *K. pneumoniae* strains, while inhibitory effects on ATCC23357 biofilm were observed when combined with 2µg/mL meropenem and various concentrations of GNS. | [22] |
| Graphene (GR) and graphene/chitosan nanoparticles (GR/CS NCs) | *K. pneumoniae* MTCC 3384 | MDR and ciprofloxacin resistance | In vitro | Treatment with 70 μg/mL of GR lead to 90% reductions in biofilm production *K. pneumoniae*, while treatment with 40 μg/mL of GR/CS NCs resulted 92% reductions in biofilm formation. | [23] |
| **Natural products derived molecules** | | | | | |
| **Plant derived molecules** | | | | | |
| Baicalein | Extended- spectrum β- lactamases positive *K. pneumoniae* | Cefotaxime resistant | In vitro | Baicalein exhibited synergistic effects on some antibiotic-resistant ESBL-positive strains of *K. pneumoniae* | [24] |
| Paeonol | *K. pneumoniae* ATCC  12657 | - | In vitro | Effective antibacterial and anti-biofilm activities against *K. pneumoniae* | [25] |
| Six natural compounds (curcumin, eugenol, linoleic acid, chitosan, reserpine and berberine) | *K. pneumoniae* clinical isolates | MDR | In vitro | 6 natural compounds could inhibit biofilm formation in high biofilm forming isolates. Among them, reserpine was the most potent biofilm inhibitors followed by linoleic acid. | [26] |
| Essential oil | *K. pneumoniae* | MDR | In vitro | Peppermint oil showed significant biofilm inhibiting ability with a 98.2% inhibition percentage, which was 95.2% for thyme oil and 77.2% for nigella oil. Thyme oil had the best biofilm eradication ability with eradication percentage from 80.1 to 98.0%, followed by peppermint and cinnamon oils | [27] |
| Essential oil compounds (EOCs) | uropathogenic New Delhi Metallo-beta-Lactamase-1 (NDM-1) producing *K. pneumoniae* | - | In vitro | Thymol, carvacrol and geraniol exhibited most promising antibacterial and antibiofilm activities. | [28]. |
| Eugenol | CRKP | Multidrug-resistant | In vitro | inhibit biofilm formation and inactivate biofilm cells | [29, 30]. |
| Tea polyphenols (TPs) | *K.*  *pneumoniae* strain CPU-K4 | - | In vitro | TPs at sub-MIC concentration resulted a drop in biofilm formation in a dose-dependent manner (23.7% inhibition at 200 μg/mL and 44.4% inhibition at 600 μg/mL). | [31] |
| Vitamin C | CR-hvKP strains KP1088 and HvKP3 | Strain KP1088 was resistant to carbapenems, cephalosporins,aminoglycosides but susceptible to polymyxins, tigecycline, and quinolones.  Strain HvKP3 was resistant to carbapenems, cephalosporins,monobactams, penicillin, aminoglycoside, quinolones and polymyxins but remained susceptible to tigecycline. | In vitro and in the mouse infection model | A dose-dependent capability to inhibit CR-hvKP growth and the biofilm formation. | [32] |
| Benzoic acid and its derivatives | *K.*  *pneumoniae* | - | In vitro | 3-hydroxy benzoic acid and 2, 5-dihydroxy benzoic acid inhibit 89-97% of biofilm formation of *K. pneumoniae* | [33] |
| **Microbial and their metabolites** | | | | | |
| CFS of *L. acidophilus* | ESBLs-producing *K. pneumoniae* clinical isolates (n=15) | The strains were  highly resistant to most tested antibiotics as ampicillin,  amoxicillin, ciprofloxacin, trimethoprim/sulfamethoxazole,  third-generation cephalosporins in a range of 66.6–100%. | In vitro | A dose-dependent antibiofilm formation activity against the fresh biofilms of *K pneumoniae;* 52%±12 of the formed biofilms were destroyed | [34] |
| *Lactobacillus* supernatants | ESBLs-producing *K. pneumoniae* (n=2) | *K. pneumoniae* 551L was resistant to cephalothin and ciprofloxacin and susceptible to gentamicin,  K. pneumoniae ATCC 700603 was resistant to cephalothin and gentamicin and susceptible to ciprofloxacin | In vitro | 95% biofilm-inhibitory and biofilm-killing properties | [35] |
| 3-Phenyllactic acid (PLA) | *K. pneumoniae* CVCC4080 | β-lactam antibiotic resistance and quinolones resistance | In vitro and mouse model | Inhibition of bacterial growth and biofilm formation. PLA could significantly increase the survival rate and reduce the histopathological injury of infected mice | [36] |
| CFS of *C. amycolatum* | *K. pneumoniae* | sensitive to ceftazidime, gentamicin, ciprofoxacin and ofoxacin, moderately sensitive to amikacin and cefepime and cefazolin*,* and resistant to imipenem | In vitro | Reduce biofilm formation of *K. pneumoniae* with the inhibition rate ranged from 3.95% to 39.69%;  destroy preformed biofilms with a rate from 7.62% to 19.32% | [37] |
| Glycolipid | *K. pneumoniae* | - | In vitro | disrupt 87% of the preformed biofilms | [38] |
| Autoinducer molecules | *K. pneumoniae* ATCC 13884 | - | In vitro | 3-methyl-2(5H)-furanone induces a biofilm inhibition of 67.38%, and the phenyl-acyl derivative 2  *0* -hydroxycinnamic acid, with an inhibition of 65.06%. | [39] |
| Sulphated polysaccharides (Cr-SPs) | *K. pneumoniae* (MTCC no. 432) | - | In vitro | Cr-SPs treatment eradicated more than 50% of preformed biofilm at 0.5 mg/mL and removed 100% biofilm at 4 mg/mL and 8 mg/mL | [40] |
| **Phage therapy** | | | | | |
| **Phages in the research stage** | | | | | |
| Phage PG14 | *K. pneumoniae* G14 | Carbapenem resistant | In vitro | 80% biofilm inhibition and 71% biofilm disruption | [41] |
| Phage vB_Kpn_ZCKp20p | *K. pneumoniae* | MDR | In vitro | Inhibit biofilm formation and disrupt the mature biofilm | [42] |
| Polyvalent phage KL-2146 | NDM producing and antibiotic-sensitive *K. pneumoniae* | Antibiotic sensitive and resistant | In vitro | Disrupt biofilms | [43] |
| Phage JKP2 | K-17 serotype specific *K. pneumoniae* | Multi-drug resistant | In vitro | Eliminate preformed biofilms with a rate of 98% for 24-hour-old biofilm, 96% of 48-hour-old biofilm, 86% and 82% for mature biofilm on day 3 and 4, respectively. | [44] |
| Phage PSKP16 | K2-Hypervirulent *K. pneumoniae* | MDR or XDR | Mouse pneumonia model | Reduce 18–64.6% of the 24-hour-old biofilms and 16.4–63.7% of the 48-hour-old biofilms.  Better therapeutic effect than gentamicin alone. | [45] |
| Recombinant depolymerase 42 | *K. pneumoniae* K47 | - | In vitro | Specific enzymatic depolymerization of *K. pneumoniae* K47 capsule and significantly inhibited biofilm formation and degraded mature biofilms. | [46] |
| Phage P510-derived depolymerase | CRKP strain KP31 | Carbapenem-resistant | In vitro | polysaccharide-degrading activity and significant antibiofilm effect | [47] |
| Bacteriophage KpV74 and phage depolymerase Dep_kpv74 | hypervirulent K2 *K. pneumoniae* 52145 | - | Mice thigh soft tissue | Bacteriophage KpV74 and phage depolymerase Dep_kpv74 are specific to lyse hypervirulent *K. pneumoniae* of the K2 capsular type, and the depolymerase Dep_kpv74 was effective against *K. pneumoniae* infection in mice thigh soft tissue, with comparable or greater efficiency than that of the bacteriophage | [48] |
| **Phage therapy used as last resort treatment clinically** | | | | | |
| KpJH46Φ2 | *K. pneumoniae* | susceptible to  ampicillin-sulbactam, ceftriaxone, ciprofloxacin, meropenem,  trimethoprim-sulfamethoxazole, and minocycline | Human prosthetic knee infection | Successfully alleviation of local symptoms and infection indicators without adverse effects with notably biofilm biomass reduction after 22 hours exposure | [49] |
| Phage vB_KpnM_M1 (M1) | *K. pneumoniae* | Pandrug-resistant | A fracture-related pandrug-resistant *K. pneumoniae* infection | The combination of phage M1 with antibiotics (meropenem and ceftazidime/avibactam) ultimately leads to the clinical resolution of the patient's infection. | [50] |

-, not mentioned

**References**

1. Moshynets, O.V., et al., *Azithromycin possesses biofilm-inhibitory activity and potentiates non-bactericidal colistin methanesulfonate (CMS) and polymyxin B against Klebsiella pneumonia.* PLoS One, 2022. **17**(7): p. e0270983.

2. She, P., et al., *SPR741, Double- or Triple-Combined With Erythromycin and Clarithromycin, Combats Drug-Resistant Klebsiella pneumoniae, Its Biofilms, and Persister Cells.* Front Cell Infect Microbiol, 2022. **12**: p. 858606.

3. Moshynets, O.V., et al., *Therapeutic Potential of an Azithromycin-Colistin Combination against XDR K. pneumoniae in a 3D Collagen-Based In Vitro Wound Model of a Biofilm Infection.* Antibiotics (Basel), 2023. **12**(2).

4. Ribera, A., et al., *Comparative Antibiofilm Efficacy of Meropenem Alone and in Combination with Colistin in an In Vitro Pharmacodynamic Model by Extended-Spectrum-β-Lactamase-Producing Klebsiella pneumoniae.* Antimicrob Agents Chemother, 2019. **63**(11).

5. Geladari, A., et al., *Dose-Dependent Synergistic Interactions of Colistin with Rifampin, Meropenem, and Tigecycline against Carbapenem-Resistant Klebsiella pneumoniae Biofilms.* Antimicrob Agents Chemother, 2019. **63**(3).

6. Ribeiro, A., et al., *In Vitro and In Vivo Synergism of Fosfomycin in Combination with Meropenem or Polymyxin B against KPC-2-Producing Klebsiella pneumoniae Clinical Isolates.* Antibiotics (Basel), 2023. **12**(2).

7. Huang, T., et al., *A Potent Antibiotic Combination of Linezolid and Polymycin B Nonapeptide Against Klebsiella pneumoniae Infection In Vitro and In Vivo.* Front Pharmacol, 2022. **13**: p. 887941.

8. Sharma, A., P. Rishi, and R. Singh, *In vitro and in vivo evaluation of DNase I in reinstating antibiotic efficacy against Klebsiella pneumoniae biofilms.* Pathog Dis, 2023. **81**.

9. Bisso Ndezo, B., C.R. Tokam Kuaté, and J.P. Dzoyem, *Synergistic Antibiofilm Efficacy of Thymol and Piperine in Combination with Three Aminoglycoside Antibiotics against Klebsiella pneumoniae Biofilms.* Can J Infect Dis Med Microbiol, 2021. **2021**: p. 7029944.

10. de la Fuente-Núñez, C., et al., *Broad-spectrum anti-biofilm peptide that targets a cellular stress response.* PLoS Pathog, 2014. **10**(5): p. e1004152.

11. Ribeiro, S.M., et al., *Antibiofilm peptides increase the susceptibility of carbapenemase-producing Klebsiella pneumoniae clinical isolates to β-lactam antibiotics.* Antimicrob Agents Chemother, 2015. **59**(7): p. 3906-12.

12. Benincasa, M., et al., *Biofilms from Klebsiella pneumoniae: Matrix Polysaccharide Structure and Interactions with Antimicrobial Peptides.* Microorganisms, 2016. **4**(3).

13. Fleeman, R.M. and B.W. Davies, *Polyproline Peptide Aggregation with Klebsiella pneumoniae Extracellular Polysaccharides Exposes Biofilm Associated Bacteria.* Microbiol Spectr, 2022. **10**(2): p. e0202721.

14. Cebrián, R., et al., *The cathelicidin-derived close-to-nature peptide D-11 sensitises Klebsiella pneumoniae to a range of antibiotics in vitro, ex vivo and in vivo.* Int J Antimicrob Agents, 2021. **58**(5): p. 106434.

15. Chatupheeraphat, C., et al., *Synergistic effect and antibiofilm activity of the antimicrobial peptide K11 with conventional antibiotics against multidrug-resistant and extensively drug-resistant Klebsiella pneumoniae.* Front Cell Infect Microbiol, 2023. **13**: p. 1153868.

16. Jeon, E., M.K. Kim, and Y. Park, *Efficacy of the bee-venom antimicrobial peptide Osmin against sensitive and carbapenem-resistant Klebsiella pneumoniae strains.* Int J Antimicrob Agents, 2023: p. 107054.

17. Farooq, U., et al., *Rifampicin conjugated silver nanoparticles: a new arena for development of antibiofilm potential against methicillin resistant Staphylococcus aureus and Klebsiella pneumoniae.* Int J Nanomedicine, 2019. **14**: p. 3983-3993.

18. Siddique, M.H., et al., *Effect of Silver Nanoparticles on Biofilm Formation and EPS Production of Multidrug-Resistant Klebsiella pneumoniae.* Biomed Res Int, 2020. **2020**: p. 6398165.

19. Scandorieiro, S., et al., *Antibiofilm Effect of Biogenic Silver Nanoparticles Combined with Oregano Derivatives against Carbapenem-Resistant Klebsiella pneumoniae.* Antibiotics (Basel), 2023. **12**(4).

20. Sharma, R.P., et al., *Assessment of antibacterial and anti-biofilm effects of zinc ferrite nanoparticles against Klebsiella pneumoniae.* Folia Microbiol (Praha), 2022. **67**(5): p. 747-755.

21. Ahmed, A., et al., *Biofilm inhibitory effect of chlorhexidine conjugated gold nanoparticles against Klebsiella pneumoniae.* Microb Pathog, 2016. **98**: p. 50-6.

22. Aguilera-Correa, J.J., et al., *Effect of Gold Nanostars Plus Amikacin against Carbapenem-Resistant Klebsiella pneumoniae Biofilms.* Biology (Basel), 2022. **11**(2).

23. Muthuchamy, M., et al., *Anti-biofilm investigation of graphene/chitosan nanocomposites against biofilm producing P. aeruginosa and K. pneumoniae.* Carbohydr Polym, 2020. **230**: p. 115646.

24. Cai, W., et al., *Synergistic effects of baicalein with cefotaxime against Klebsiella pneumoniae through inhibiting CTX-M-1 gene expression.* BMC Microbiol, 2016. **16**(1): p. 181.

25. Qian, W., et al., *Antibacterial and anti-biofilm activities of paeonol against Klebsiella pneumoniae and Enterobacter cloacae.* Biofouling, 2021. **37**(6): p. 666-679.

26. Magesh, H., et al., *Identification of natural compounds which inhibit biofilm formation in clinical isolates of Klebsiella pneumoniae.* Indian J Exp Biol, 2013. **51**(9): p. 764-72.

27. Mohamed, S.H., et al., *Combination of essential oil and ciprofloxacin to inhibit/eradicate biofilms in multidrug-resistant Klebsiella pneumoniae.* J Appl Microbiol, 2018. **125**(1): p. 84-95.

28. Kwiatkowski, P., et al., *Antibacterial and Anti-Biofilm Activities of Essential Oil Compounds against New Delhi Metallo-beta-Lactamase-1-Producing Uropathogenic Klebsiella pneumoniae Strains.* Antibiotics (Basel), 2022. **11**(2).

29. Qian, W., et al., *Antimicrobial activity of eugenol against carbapenem-resistant Klebsiella pneumoniae and its effect on biofilms.* Microb Pathog, 2020. **139**: p. 103924.

30. Liu, W., et al., *Eugenol eliminates carbapenem-resistant Klebsiella pneumoniae via reactive oxygen species mechanism.* Front Microbiol, 2023. **14**: p. 1090787.

31. Liu, W., et al., *Tea polyphenols inhibits biofilm formation, attenuates the quorum sensing-controlled virulence and enhances resistance to Klebsiella pneumoniae infection in Caenorhabditis elegans model.* Microb Pathog, 2020. **147**: p. 104266.

32. Xu, C., et al., *Bactericidal, anti-biofilm, and anti-virulence activity of vitamin C against carbapenem-resistant hypervirulent Klebsiella pneumoniae.* iScience, 2022. **25**(3): p. 103894.

33. Rohatgi, A. and P. Gupta, *Benzoic acid derivatives as potent antibiofilm agents against Klebsiella pneumoniae biofilm.* J Biosci Bioeng, 2023. **136**(3): p. 190-197.

34. El-Mokhtar, M.A., et al., *Antagonistic Activities of Cell-Free Supernatants of Lactobacilli Against Extended-Spectrum β-Lactamase Producing Klebsiella pneumoniae and Pseudomonas aeruginosa.* Infect Drug Resist, 2020. **13**: p. 543-552.

35. Kheiri, F., R.K. Kermanshahi, and M.M. Feizabadi, *The Inhibitory Effects of Lactobacillus Supernatants and Their Metabolites on the Growth and Biofilm Formation of Klebsiella pneumoniae.* Infect Disord Drug Targets, 2020. **20**(6): p. 902-912.

36. Yu, J., et al., *Antimicrobial activity of phenyllactic acid against Klebsiella pneumoniae and its effect on cell wall membrane and genomic DNA.* Braz J Microbiol, 2023. **54**(4): p. 3245-3255.

37. Gladysheva, I.V. and S.V. Cherkasov, *Antibiofilm activity of cell-free supernatants of vaginal isolates of Corynebacterium amycolatum against Pseudomonas aeruginosa and Klebsiella pneumoniae.* Arch Microbiol, 2023. **205**(4): p. 158.

38. Gharaei, S., et al., *Isolation, Optimization, and Structural Characterization of Glycolipid Biosurfactant Produced by Marine Isolate Shewanella algae B12 and Evaluation of Its Antimicrobial and Anti-biofilm Activity.* Appl Biochem Biotechnol, 2022. **194**(4): p. 1755-1774.

39. Cadavid, E. and F. Echeverri, *The Search for Natural Inhibitors of Biofilm Formation and the Activity of the Autoinductor C6-AHL in Klebsiella pneumoniae ATCC 13884.* Biomolecules, 2019. **9**(2).

40. Vishwakarma, J., et al., *Algal polysaccharide's potential to combat respiratory infections caused by Klebsiella pneumoniae and Serratia marcescens biofilms.* Appl Biochem Biotechnol, 2022. **194**(2): p. 671-693.

41. Mulani, M.S., S.N. Kumkar, and K.R. Pardesi, *Characterization of Novel Klebsiella Phage PG14 and Its Antibiofilm Efficacy.* Microbiol Spectr, 2022. **10**(6): p. e0199422.

42. Zaki, B.M., et al., *Characterization and comprehensive genome analysis of novel bacteriophage, vB_Kpn_ZCKp20p, with lytic and anti-biofilm potential against clinical multidrug-resistant Klebsiella pneumoniae.* Front Cell Infect Microbiol, 2023. **13**: p. 1077995.

43. Gilcrease, E.B., et al., *A Klebsiella pneumoniae NDM-1+ bacteriophage: Adaptive polyvalence and disruption of heterogenous biofilms.* Front Microbiol, 2023. **14**: p. 1100607.

44. Asif, M., et al., *A K-17 serotype specific Klebsiella phage JKP2 with biofilm reduction potential.* Virus Res, 2023. **329**: p. 199107.

45. Rahimi, S., et al., *Characterization of novel bacteriophage PSKP16 and its therapeutic potential against β-lactamase and biofilm producer strain of K2-Hypervirulent Klebsiella pneumoniae pneumonia infection in mice model.* BMC Microbiol, 2023. **23**(1): p. 233.

46. Wu, Y., et al., *A Novel Polysaccharide Depolymerase Encoded by the Phage SH-KP152226 Confers Specific Activity Against Multidrug-Resistant Klebsiella pneumoniae via Biofilm Degradation.* Front Microbiol, 2019. **10**: p. 2768.

47. Li, M., et al., *Identification of a phage-derived depolymerase specific for KL64 capsule of Klebsiella pneumoniae and its anti-biofilm effect.* Virus Genes, 2021. **57**(5): p. 434-442.

48. Pertics, B.Z., T. Kovács, and G. Schneider, *Characterization of a Lytic Bacteriophage and Demonstration of Its Combined Lytic Effect with a K2 Depolymerase on the Hypervirulent Klebsiella pneumoniae Strain 52145.* Microorganisms, 2023. **11**(3).

49. Cano, E.J., et al., *Phage Therapy for Limb-threatening Prosthetic Knee Klebsiella pneumoniae Infection: Case Report and In Vitro Characterization of Anti-biofilm Activity.* Clin Infect Dis, 2021. **73**(1): p. e144-e151.

50. Eskenazi, A., et al., *Combination of pre-adapted bacteriophage therapy and antibiotics for treatment of fracture-related infection due to pandrug-resistant Klebsiella pneumoniae.* Nat Commun, 2022. **13**(1): p. 302.
